# Children’s Pronoun Interpretation Problems Are Related to Theory of Mind and Inhibition, But Not Working Memory

**DOI:** 10.3389/fpsyg.2021.610401

**Published:** 2021-06-04

**Authors:** Sanne J. M. Kuijper, Catharina A. Hartman, Petra Hendriks

**Affiliations:** ^1^Inclusive and Special Needs Education Unit, Department of Pedagogical and Educational Sciences, University of Groningen, Groningen, Netherlands; ^2^Department of Psychiatry, Interdisciplinary Center Psychopathology and Emotion Regulation (ICPE), University of Groningen, University Medical Center Groningen, Groningen, Netherlands; ^3^Center for Language and Cognition Groningen, University of Groningen, Groningen, Netherlands

**Keywords:** ADHD, autism, inhibition, language acquisition, perspective taking, syntax, theory of mind, working memory

## Abstract

In several languages, including English and Dutch, children’s acquisition of the interpretation of object pronouns (e.g., *him*) is delayed compared to that of reflexives (e.g., *himself*). Various syntactic and pragmatic explanations have been proposed to account for this delay in children’s acquisition of pronoun interpretation. This study aims to provide more insight into this delay by investigating potential cognitive mechanisms underlying this delay. Dutch-speaking children between 6 and 12 years old with autism spectrum disorder (ASD; *n* = 47), attention-deficit/hyperactivity disorder (ADHD; *n* = 36) or typical development (TD; *n* = 38) were tested on their interpretation and production of object pronouns and reflexives and on theory of mind, working memory, and response inhibition. It was found that all three groups of children had difficulty with pronoun interpretation and that their performance on pronoun interpretation was associated with theory of mind and inhibition. These findings support an explanation of object pronoun interpretation in terms of perspective taking, according to which listeners need to consider the speaker’s perspective in order to block coreference between the object pronoun and the subject of the same sentence. Unlike what is predicted by alternative theoretical accounts, performance on pronoun interpretation was not associated with working memory, and the children made virtually no errors in their production of object pronouns. As the difficulties with pronoun interpretation were similar for children with ASD, children with ADHD and typically developing children, this suggests that certain types of perspective taking are unaffected in children with ASD and ADHD.

## Introduction

A fundamental aspect of children’s language acquisition is learning what the linguistic expressions in their language refer to. Proper names (e.g., *John*) generally have a fixed reference. In contrast, personal pronouns (e.g., *he*, *she*, *him*, *her*) and reflexives (e.g., *himself*, *herself*) depend on other words in the sentence or the discourse for their interpretation. For instance, in the sentence “Paul got upset when John accidentally hit him” the object pronoun *him* refers back to the subject of the previous clause, *Paul*. The fact that *him* cannot refer back to the subject of the same clause, *John*, indicates that not only the linguistic discourse, but also grammatical principles play a role. These grammatical principles also apply to reflexives, such as *himself*, which must refer back to the subject of the same clause and cannot refer back to the subject of a previous clause. The patterns of use and interpretation of pronouns and reflexives have been the focus of much theoretical work in linguistics, including Chomsky’s syntactic binding theory (Chomsky, 1981), later revisions of binding theory such as Reinhart and Reuland’s reflexivity account (Reinhart and Reuland, 1993) and Reuland’s primitives of binding account (Reuland, 2011), and pragmatic alternatives to binding theory such as Levinson (1991, 2000).

Already early on it was realized that language acquisition research can inform linguistic theorizing (e.g., Chien and Wexler, 1990). In Chomsky’s original conception of binding theory, the use and interpretation of pronouns and reflexives is governed by two related principles of the grammar: Roughly speaking, Principle A requires reflexives in simple transitive sentences to refer to the same referent as the subject (resulting in a so-called coreferential interpretation), and Principle B requires pronouns not to corefer with the subject. It is thus expected that children would show mastery of pronouns and reflexives at more or less the same moment in their language development. However, language acquisition research revealed that children’s interpretation of object pronouns in English is delayed in comparison to their interpretation of reflexives (e.g., Chien and Wexler, 1990; Grimshaw and Rosen, 1990). For example, in a situation in which two referents are present in the discourse, children until the age of 6 incorrectly allow the object pronoun in sentences like (1) to corefer with the subject. At the same time, they correctly interpret reflexives such as in (2) as coreferring with the subject from age 4.

(1) The elephant is hitting him.
(2) The elephant is hitting himself.

This phenomenon is known as the Delay of Principle B Effect, or Pronoun Interpretation Problem. Only around the age of 10 or 11 years old, children’s performance on object pronoun interpretation is adult-like (Philip and Coopmans, 1996; Başkent et al., 2013).

In English, the pronoun *him* and the reflexive *himself* are quite similar in form. In contrast, the Dutch pronoun *hem* (‘him’) and the Dutch reflexive *zichzelf* (‘himself/herself’) are clearly distinct forms. Nevertheless, the Pronoun Interpretation Problem is also observed in Dutch (e.g., Philip and Coopmans, 1996; Spenader et al., 2009; van Rij et al., 2010). This indicates that the Pronoun Interpretation Problem is not caused by children’s confusion of the two forms. Whereas the Pronoun Interpretation Problem occurs in children’s typical acquisition of English, Dutch and several other languages, it does not occur in all languages and for example is absent in Romance languages. Thus, the Pronoun Interpretation Problem is not a universal phenomenon in language acquisition but rather appears to depend on certain grammatical properties of the language. As yet, no satisfactory explanation has been given for this cross-linguistic variation, since it is not clear what properties the languages have in common that show or do not show a Pronoun Interpretation Problem. For example, whereas English and Dutch show the Pronoun Interpretation Problem, the closely related language German does not (Ruigendijk, 2008; see also Ruigendijk et al., 2010); as such, German patterns with the Romance languages, which differ from German in that they have clitic pronouns. Although this cross-linguistic variation in the Pronoun Interpretation Problem is relevant for generalizing the findings of the present study, the present study focuses on Dutch with the aim to shed more light on the interaction between grammar and cognitive processes in pronoun interpretation in languages that show a Pronoun Interpretation Problem.

### Explaining the Pronoun Interpretation Problem

In the linguistic literature, various explanations have been put forward for the Pronoun Interpretation Problem. The three explanations most relevant for the current study are discussed below, namely the pragmatic explanation, the working memory explanation, and the perspective taking explanation. These explanations all assume that the interpretation of reflexives is fully determined by the grammar, but that the interpretation of pronouns requires some additional process: pragmatics, reference-set-computation, or bidirectional optimization.

Chien and Wexler (1990) argue that children possess the relevant grammatical knowledge of the binding principles required for a mature interpretation of object pronouns and reflexives (cf. Chomsky, 1981), but still lack the pragmatic skills for their mature usage in context (cf. Thornton and Wexler, 1999). Chien and Wexler’s (1990) pragmatic explanation is based on a distinction between syntactic binding (e.g., the relation between the reflexive *himself* and the quantified subject *every elephant* in the sentence “Every elephant is hitting himself”) and pragmatic coreference (e.g., the relation between the pronoun *he* and its non-local referential antecedent *an elephant* in the sentence pair “There is an elephant. He is large”). According to their explanation, children have knowledge of the restrictions on syntactic binding but have difficulty with the restrictions on pragmatic coreference. In particular, Chien and Wexler refer to so-called ‘accidental coreference’ as a source of confusion for children. Accidental coreference occurs when the object pronoun and the referential subject of the sentence accidentally refer to the same individual (as *he* and *him* do in “That must be John. At least he looks like him”), despite the fact that this is disallowed by the binding principles. Accidental coreference is only possible in certain (rare) contexts. To explain why English-speaking children show a Pronoun Interpretation Problem, Chien and Wexler (1990) argue that children have pragmatic difficulty with distinguishing between contexts in which accidental coreference is permitted and contexts in which it is not. Crucially, accidental coreference is not allowed in sentences like (1), but children may not yet have knowledge of this pragmatic restriction.

Under the view that children’s errors with object pronouns are due to their confusion about accidental coreference, children should also show problems in pronoun production and use object pronouns to express a coreferential meaning in all contexts, so also in contexts in which accidental coreference is not allowed. However, English-speaking children between 2;3 and 3;1 years old already produce object pronouns correctly in their spontaneous speech (Bloom et al., 1994) and English- and Dutch-speaking children’s production of object pronouns in an experimental setting was found to be adult-like from age 4;6 (for English: De Villiers et al., 2006; Matthews et al., 2009; for Dutch: Spenader et al., 2009). This makes an explanation in terms of lack of pragmatic skills unlikely. Additionally, the distinction Chien and Wexler (1990) found between children’s pronoun interpretation in syntactic binding environments and pragmatic coreference environments has been questioned by later studies as an artifact of their experimental materials (e.g., Elbourne, 2005; Conroy et al., 2009).

More recent explanations of the Pronoun Interpretation Problem attribute this problem to children’s limited processing resources (e.g., Reinhart, 2006, 2011; Ruigendijk et al., 2011). For example, Reinhart (2011) argues that the Pronoun Interpretation Problem results from children’s insufficient working memory capacity (see also Grodzinsky and Reinhart, 1993; Montgomery and Evans, 2009). According to Reinhart (2011), there are two means by which object pronouns can be interpreted: by syntactic binding and by pragmatic coreference. If the grammar allows two interpretational possibilities, the process of reference-set computation is required (Reinhart, 2006, 2011). Reference-set computation compares the different structures and their interpretations, and discards an interpretation if there is a more economical way to obtain that interpretation. Adults use reference-set computation to block pragmatic coreference between an object pronoun and the local subject, as pragmatic coreference is assumed to be a less economical way to express a coreferential interpretation than syntactic binding. Reinhart claims that children have insufficient working memory to perform this costly computation and therefore resort to guessing in their interpretation of object pronouns (Reinhart, 2011). Reference-set computation does not apply in production, since speakers already know which meaning they want to express. Therefore, children’s production of object pronouns is predicted to be adult-like (Reinhart, 2006).

Another explanation of the Pronoun Interpretation Problem linking this problem to children’s cognitive limitations is proposed by Hendriks and Spenader (2006). They argue that the Pronoun Interpretation Problem is caused by core properties of the grammar itself. Instead of formulating their account in terms of universally valid syntactic principles, as Chien and Wexler (1990) and Reinhart (2006, 2011) do, they formulate their account in terms of violable constraints that differ in strength, as in Optimality Theory (Prince and Smolensky, 2004). The optimal form or meaning is the form or meaning that satisfies the constraints of the grammar best. The constraints determine, for a given input, what is the optimal output for that input in production (when the input is a meaning and the output is a form) or comprehension (when the input is a form and the output is a meaning). As the constraints of the grammar are sensitive to whether they evaluate forms or meanings, they may yield a different form-meaning mapping in comprehension than in production (Smolensky, 1996). To achieve communicative success in spite of these potentially different outcomes in production and comprehension, it has been argued that production and comprehension must be taken into account simultaneously in determining the mature pattern of forms and meanings, through a procedure known as bidirectional optimization (e.g., de Hoop and Krämer, 2006; Legendre et al., 2016). This procedure of bidirectional optimization can be seen as the formalization, within the grammar, of the process of perspective taking (Hendriks, 2014).

In Hendriks and Spenader’s (2006) constraint-based account, the constraints of the grammar select both a coreferential and a non-coreferential interpretation as the optimal meaning for an object pronoun, resulting in ambiguity for this pronoun. When encountering an object pronoun, adult listeners are able to block the coreferential interpretation for the pronoun by taking into account the perspective of the speaker: if the speaker would have wanted to express a coreferential interpretation, the speaker would have used a reflexive instead of a pronoun. Since the speaker did not use a reflexive, the speaker must have intended to express a non-coreferential interpretation. Young children are argued to not yet be able to take into account the perspective of the speaker in their interpretation of object pronouns in a consistent way. Hence, they consider pronouns to be ambiguous, thus showing the Pronoun Interpretation Problem. Such perspective taking is expected to require theory of mind abilities (Hendriks, 2014). Indeed, first-order theory of mind is generally acquired well before children show adult-like performance on pronoun interpretation (De Villiers et al., 2006). Furthermore, perspective taking may also require inhibition skills, since the listener must suppress the coreferential meaning in order to select the correct non-coreferential meaning for the pronoun. In Hendriks and Spenader’s constraint-based account, the same constraints giving rise to ambiguity of object pronouns in comprehension result in the correct interpretation of reflexives in comprehension and the correct selection of a pronoun or reflexive in production. Thus, children’s production of object pronouns is predicted to be adult-like.

The role of inhibition is not only compatible with the perspective-taking explanation, but in principle follows from all accounts of pronoun processing that assume several potential antecedents for the pronoun to be activated during initial stages of processing and assume the grammatical antecedent to compete with binding theory-incompatible antecedents (e.g., Badecker and Straub, 2002; Clackson et al., 2011). Inhibition is needed to subsequently suppress the antecedent that is incompatible with the binding principles. This contrasts with so-called initial-filter models of pronoun processing, that assume that the principles of binding theory are applied early during sentence processing and act as an initial filter, immediately ruling out antecedents that are not compatible with the binding principles (Nicol and Swinney, 1989).

In sum, while the pragmatic explanation attributes children’s pronoun interpretation problems to their lack of pragmatic knowledge and predicts that children also make errors with pronouns in production, Reinhart’s explanation based on reference-set computation predicts that errors in pronoun interpretation are caused by insufficient working memory, and Hendriks and Spenader’s explanation based on bidirectional optimization predicts that these errors result from a failure to take into account the speaker’s perspective, which requires theory of mind abilities and inhibition skills.

### Language in Children With ASD and Children With ADHD

The present study aims to clarify how children acquire object pronoun interpretation and production by investigating the role of three possible underlying cognitive mechanisms in pronoun interpretation and production, namely working memory, theory of mind, and inhibition. We designed our study in such a way that we maximized the variation in cognitive mechanisms as well as outcome measures by including children with autism spectrum disorder (ASD), children with attention-deficit/hyperactivity disorder (ADHD) and a group of typically developing (TD) children in our sample. Children with ASD are known to have difficulties in social interaction and communication and show restricted, repetitive behaviors and interests (DSM-5, American Psychiatric Association, 2013). Children with ADHD show a persistent pattern of inattention and/or hyperactivity–impulsivity (DSM-5, American Psychiatric Association, 2013).

Problems in theory of mind have been frequently reported in children with ASD (e.g., Baron-Cohen et al., 1993) and sometimes in children with ADHD (Buitelaar et al., 1999; Kuijper et al., 2015, 2017; Mary et al., 2016). Furthermore, working memory problems and problems in inhibition have been reported in children with ADHD and children with ASD (e.g., Pennington and Ozonoff, 1996; Nydén et al., 2001; Geurts et al., 2004b; Hill, 2004; Martinussen et al., 2005; Happé et al., 2006; Schmitt et al., 2018; Habib et al., 2019). Thus, the deficits in social and cognitive functioning found in children with ASD partly overlap with those in children with ADHD (Bishop and Baird, 2001; Nijmeijer et al., 2010; Rommelse et al., 2011; Demopoulos et al., 2013; Johnson et al., 2015).

Besides difficulties with theory of mind, working memory, and inhibition, both children with ASD and children with ADHD exhibit problems with language and communication. Pragmatic problems are among the core deficits of ASD (DSM-5, American Psychiatric Association, 2013). While the pragmatic deficits in ASD are well documented, less is known about problems in ASD with the structural, or morphosyntactic, properties of language. Some studies did not find morphosyntactic impairments in children with ASD (Bartolucci et al., 1976; Tager-Flusberg, 1981). In contrast, other studies found evidence for morphosyntactic impairments or delays in (subgroups of) children with ASD (Kjelgaard and Tager-Flusberg, 2001; Eigsti et al., 2007; Durrleman et al., 2017). These results indicate that there is considerable heterogeneity in language impairments in ASD (for an overview, see Boucher, 2012).

In ADHD, language deficits are not part of the diagnosis. However, recent studies using parental and teacher questionnaires suggest that in children with ADHD pragmatic use of language is often impaired (for an overview, see Green et al., 2014). Most studies investigating language impairments in ADHD did not find morphosyntactic impairments in children with ADHD (e.g., Kim and Kaiser, 2000; Geurts et al., 2004a; Geurts and Embrechts, 2008; Helland et al., 2012), but some did (Oram et al., 1999; Papaeliou et al., 2015). The language and communication problems of children with ADHD may therefore partly overlap with those observed in children with ASD (e.g., Geurts and Embrechts, 2008).

Although the findings on morphosyntactic impairments of children with ASD and ADHD are equivocal, it may well be that children with ASD or ADHD experience a greater delay in object pronoun interpretation than typically developing children, due to cognitive deficits. Perovic et al. (2013), however, found that high-functioning children with ASD and TD children demonstrated similar difficulties in their comprehension of object pronouns in English. To our knowledge, object pronoun interpretation has not been investigated yet in children with ADHD. The production of object pronouns has been studied in ASD, but mainly in languages such as French and Greek that have clitic pronouns occurring in a special position to the immediate left of the verb (e.g., Terzi et al., 2014; Tuller et al., 2017; Prévost et al., 2018). This contributes to the complexity of the construction and may explain the difficulty these children have with the production of clitic object pronouns. Thus, in addition to our main aim of investigating possible cognitive mechanisms underlying the Pronoun Interpretation Problem, our study will also yield further insight into the relation between pronoun comprehension and pronoun production in children with ASD and children with ADHD.

In our study we focus on children in the age range of 6–12 years, as in this age range in TD children the Pronoun Interpretation Problem gradually decreases (Başkent et al., 2013). Therefore, we expect most variation in object pronoun interpretation performance in this age range. To investigate possible cognitive mechanisms underlying the interpretation of object pronouns, we administer a theory of mind task, a working memory task, and an inhibition task. Following Hendriks and Spenader (2006), object pronoun interpretation is expected to be associated with theory of mind and inhibition. Alternatively, following Reinhart’s (2011) account, object pronoun interpretation is hypothesized to be associated with working memory.

## Materials and Methods

### Participants

In total 126 Dutch-speaking children were tested (51 with ASD, 37 with ADHD, and 38 TD children), ranging in age from 6;1 to 12;10 (*M* = 9;1, *SD* = 1;9).

#### Children With ASD

Children in the ASD group were diagnosed with Autistic Disorder (*n* = 10), PDD-NOS (*n* = 34) or Asperger’s Disorder (*n* = 7) by independent clinicians on the basis of the DSM-IV-TR criteria (American Psychiatric Association, 2000). Additional inclusion criteria were that the children had a Full Scale Intelligence Quotient (FSIQ) above 75 and verbal communication skills. Furthermore, both the Autism Diagnostic Interview Revised (ADI-R: Rutter et al., 2003) and the Autism Diagnostic Observation Schema (ADOS, Lord et al., 1999) were administered by certified psychologists. Children in this study were included in the ASD group if they met the ADOS criteria for autism or ASD and/or the ADI-R criteria for autism or ASD (cf. Risi et al.’s ASD2 criteria, Risi et al., 2006). Three children from the ASD group were excluded from further analysis because they did not meet these criteria. One more child was excluded later because he finished neither the pronoun and reflexive comprehension task nor the production task (see section “Procedure”), leaving 47 children in the ASD group. To document the extent to which ADHD symptoms were present, the Parent Interview for Child Symptoms (PICS: Ickowicz et al., 2006) was administered. Seven children in the ASD group scored above the ADHD cut-offs on the PICS (see Table 1). In line with their clinical ASD diagnosis, we included these children in the ASD group.

**TABLE 1:** Mean scores (standard deviations) of age, clinical interviews, WISC-III, PPVT, False Belief task, n-back task, and stop task.

	**ASD (*n* = 47)**		**ADHD (*n* = 36)**		**TD (*n* = 38)**		**Group differences (Bonferroni corrected *post hoc* analyses)**
	***M***	**(*SD*)**	***M***	**(*SD*)**	***M***	**(*SD*)**	
% Male	87		83		66		
Age	9.3	(1.10)	8.9	(1.7)	9.0	(1.9)	n.s.
ADI-R^1^							
Social Interaction	16.40	(6.06)	4.58	(4.10)	1.82	(3.09)	ASD^∗∗∗^ > ADHD > TD^∗^
Communication	12.62	(4.38)	4.03	(2.68)	1.34	(1.55)	ASD^∗∗∗^ > ADHD > TD^∗∗^
Stereotyped Behavior	4.40	(2.59)	1.42	(1.56)	0.32	(0.66)	ASD^∗∗∗^ > ADHD > TD^∗^
Behavior < 3 years	3.00	(0.98)	1.47	(1.54)	0.13	(0.41)	ASD^∗∗∗^ > ADHD > TD^∗∗∗^
ADOS module 3^2^							
Communication	2.67	(1.43)	1.09	(0.92)	0.53	(0.76)	ASD^∗∗∗^ > ADHD, TD
Social interaction	7.26	(3.12)	2.57	(1.96)	1.50	(1.72)	ASD^∗∗∗^ > ADHD, TD
Com + Soc	9.93	(4.17)	3.66	(2.57)	2.03	(1.99)	ASD^∗∗∗^ > ADHD, TD
RRB	1.13	(1.24)	0.29	(0.57)	0.16	(0.44)	ASD^∗∗∗^ > ADHD, TD
Social Affect	8.89	(4.19)	2.83	(2.36)	1.74	(2.02)	ASD^∗∗∗^ > ADHD, TD
SA + RRB	10.02	(4.68)	3.11	(2.37)	1.89	(2.15)	ASD^∗∗∗^ > ADHD, TD
PICS^3^							
Inattention	2.26	(2.07)	3.61	(2.18)	0.11	(0.39)	ADHD^∗∗^ > ASD > TD^∗∗∗^
Hyperactivity/impulsivity	1.98	(1.97)	5.22	(2.45)	0.29	(0.57)	ADHD^∗∗∗^ > ASD > TD^∗∗∗^
WISC-III							
Block Design	9.87	(3.57)	8.33	(3.02)	11.16	(3.23)	ADHD < TD^∗∗^
Vocabulary	8.81	(3.18)	9.44	(2.10)	11.82	(2.51)	ASD^∗∗∗^, ADHD^∗∗^ < TD
Estimated Full scale IQ	96.19	(17.47)	93.26	(12.80)	109.02	(13.64)	ASD, ADHD < TD^∗∗∗^
PPVT							
WBQ	104.85	(14.33)	99.97	(12.57)	108.84	(10.72)	ADHD < TD^∗^
False Belief Task							
Proportion correct FB1	0.89	(0.19)	0.88	(0.14)	0.94	(0.11)	n.s.
Proportion correct FB2	0.56	(0.40)	0.55	(0.34)	0.78	(0.29)	ASD, ADHD < TD^∗^
N-Back Task							
Number correct 2back	39.02	(7.95)	38.19	(7.45)	41.77	(5.28)	n.s.
Stop Task							
SSRT	257.39	(96.51)	254.84	(94.25)	256.74	(77.59)	n.s.

*p < 0.05; **p < 0.01; ***p < 0.001. n.s., non-significant.

*^1^Five children in the ADHD group scored on the ADI-R above the cut-off for ASD (on the basis of Risi et al.’s criteria, Risi et al., 2006).*

*^2^Two children in the ADHD group scored above the ADOS criteria for ASD.*

*^3^Seven children in the ASD group scored within our criteria for ADHD on the PICS (above or one point below the cut-off on the PICS).*

#### Children With ADHD

Children in the ADHD group were diagnosed with Combined type (*n* = 19), Predominantly Hyperactive-Impulsive type (*n* = 12) or Predominantly Inattentive type (*n* = 6) by independent clinicians on the basis of the DSM-IV-TR criteria (American Psychiatric Association, 2000). Furthermore, both the Parent Interview for Child Symptoms (PICS: Ickowicz et al., 2006) and the Teacher Telephone Interview-IV (TTI-IV: Tannock et al., 2002) were administered by trained clinicians. Six children with ADHD lacked TTI information. Four of them already scored above the cut-off for ADHD based on parent information alone. The remaining two children scored 1 point below the cut-off for ADHD. Since these children scored comparable on the PICS to the other children in the ADHD group (for whom TTI scores combined with their PICS scores reached the cut-off), we included them in the analyses. Seven children in the ADHD group scored within ASD criteria on the ADOS or ADI-R (see Table 1). In line with their clinical diagnosis, we included these children in the ADHD group. One child was excluded later for task-related reasons (see section “Procedure”), leaving 36 children in the ADHD group.

#### TD Children

Children in the TD group had not been diagnosed with ASD or ADHD. The ADOS, ADI-R and PICS were administered by trained clinicians in this group as well. None of the children scored above the cut-offs for ASD or ADHD described above.

### Materials

#### Background Variables

IQ of the children was assessed by two subtests (Vocabulary and Block Design) of the Dutch Wechsler Intelligence Scale for Children (WISC-III NL: Kort et al., 2002). Verbal ability was assessed by the Dutch version of the Peabody Picture Vocabulary Test-III (PPVT: Dunn and Dunn, 1997; Schlichting, 2005). Group means and standard deviations for age, IQ, PPVT, and clinical interviews can be found in Table 1.

#### Pronoun and Reflexive Comprehension Task

**FIGURE 1:**
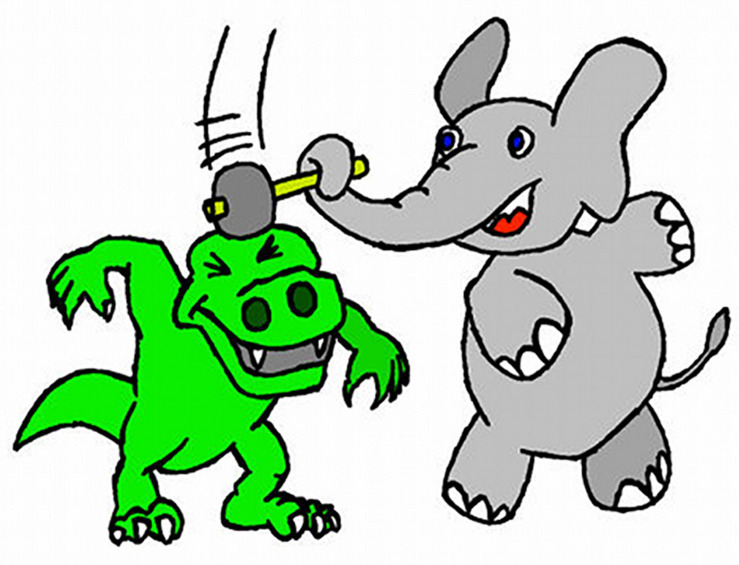
Example of picture showing an other-oriented action.

**FIGURE 2:**
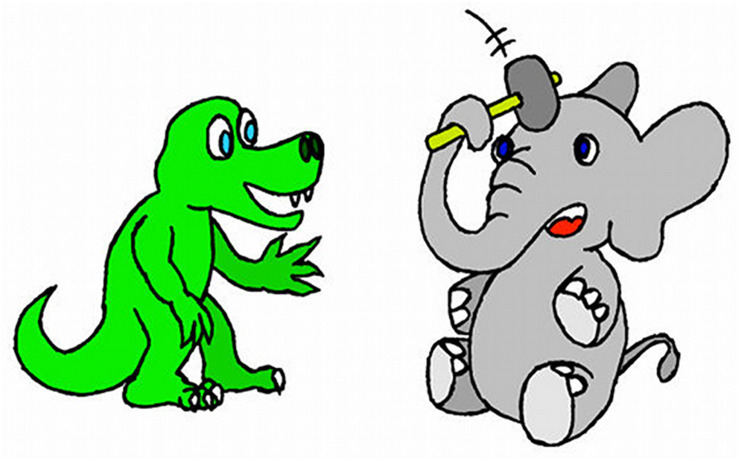
Example of picture showing a self-oriented action.

To test the comprehension of object pronouns and reflexives, we carried out a Picture Verification Task. Children saw one picture at a time. The picture showed two animals engaged in an other-oriented action (Figure 1) or a self-oriented action (Figure 2).

At the same time, the child heard an introductory sentence, followed by a test sentence with either an object pronoun or a reflexive [see example (3) and (4)].

(3) Introductory sentence:
Een krokodil en een olifant zijn op de stoep.
‘An alligator and an elephant are on the sidewalk.’
(4) Test sentence:
De olifant slaat hem/zichzelf.
‘The elephant is hitting him/himself.’

The materials were based on the materials of Spenader et al. (2009) and van Rij et al. (2010). The transitive verbs that were used in the test sentences were the Dutch translations of *to tickle*, *to hit*, *to bite*, *to point to*, *to draw*, *to paint*, *to tie*, *to make up*, and *to dress*. The child was asked whether or not the recorded sentence matched the picture. Children had to respond by pressing the yes-key when the sentence matched the picture, and by pressing the no-key when the sentence did not match the picture. On trials for which the children decided that the sentence did not match the picture, they were asked to explain why. A second tester noted these justifications.

The task started with two practice items to determine whether the children understood the task. The comprehension task consisted of 34 items: 2 practice items, 16 test items (eight items in the reflexive condition and eight items in the pronoun condition), and 16 control items without an object pronoun or reflexive. The control items were included to measure children’s general understanding of the task. In half of the items the sentence matched the picture (match condition). In the other half of the items the sentence and the picture did not match (mismatch condition). Mismatch items contained either a picture of an other-oriented action in combination with a sentence with a reflexive, or a picture of a self-oriented action in combination with a sentence with a pronoun.

We expect children exhibiting the Pronoun Interpretation Problem to make more errors in the pronoun mismatch condition than in the pronoun match condition (cf. Chien and Wexler, 1990; van Rij et al., 2010). Because these children are expected to allow both interpretations of the object pronoun, they will correctly accept the non-coreferential interpretation in the match condition, but also incorrectly accept the coreferential interpretation in the mismatch condition, leading to lower performance on the mismatch condition than on the match condition. Furthermore, we expect these children to not make errors in the reflexive condition if they are not impaired in their syntactic abilities. The reflexive conditions (match and mismatch) thus serve as control conditions to measure children’s mastery of the syntactic knowledge required for interpreting the test sentences.

#### Pronoun and Reflexive Production Task

To check whether children’s production of object pronouns and reflexives is adult-like in the same sentence context that is used in the comprehension task, we carried out a sentence elicitation task. This production task was designed to be similar to the comprehension task, as it is well-known from the literature on linguistic reference that pronoun interpretation and use is highly dependent on contextual features such as the structure of the linguistic discourse and visual information. This also holds for object pronouns in simple transitive sentences, which were used in the comprehension task. For example, Dutch-speaking children’s as well as adults’ online processing of object pronouns in simple transitive sentences is influenced by the linear order in which the potential antecedents of the pronoun are mentioned in the preceding sentence (van Rij et al., 2016). To rule out the possibility that observed differences between production and comprehension outcomes are caused by subtle differences in verbal or visual materials or task instructions, we kept the two tasks as similar as possible. Thus, the production task allows us to test whether the children obey the binding principles in production.

The visual materials of the production task were based on the materials of Spenader et al. (2009). Pictures that were used in the production task were similar to those in the comprehension task. When a picture with an other-oriented action was used in the comprehension task, the corresponding picture with the self-directed action was used in the production task and vice versa. In this way, no picture was shown in both comprehension and production, to avoid possible priming effects. The production task consisted of 16 items in total: two practice items and 14 test items. No filler items were used. Half of the items displayed an other-oriented action, the other half a self-oriented action.

Children saw one picture at a time. They were instructed to first introduce both animals and then to describe the action, leading to sentences like “I see an elephant and a crocodile. The elephant is hitting himself.” The production task started with two practice items to determine whether the children understood the task, before they were presented with the test items.

#### Theory of Mind

To test theory of mind, we used a second-order False Belief task adopted from Hollebrandse et al. (2014). False Belief tasks require one to understand that another person has his or her own beliefs and that these can be different from one’s own beliefs (e.g., Baron-Cohen et al., 1985). The task measured both first-order False Belief (FB) (involving the belief of another person) and second-order FB (involving the belief of another person about someone else’s belief). We used Hollebrandse et al.’s (2014) verbal rather than their low-verbal second-order FB task for this study, as their low-verbal task turned out to be much more demanding for children in the age range tested than their verbal task, for reasons unrelated to theory of mind abilities (see Hollebrandse et al., 2014, for discussion). As most typically developing children pass first-order FB tasks around age 4 (see the meta-analysis of Wellman et al., 2001), and our participant group is between 6 and 12 years old, we expect ceiling performance on the first-order FB questions. Therefore, of specific interest to our study is children’s performance on the second-order FB questions.

Each story in the FB task starts with an initial belief that is shared by the two main characters in the story (e.g., Sam and Maria both believe that they are selling cookies at the bake sale). This belief changes in the middle of the story for the first character without the second character knowing about this (e.g., while Maria has gone out to buy cookies, Sam hears that they are selling apple pie instead), and next changes for the second character without the first character knowing about this (e.g., Maria finds out at the bake sale that they are only selling waffles, without Sam knowing about this). As a result, the story involves three distinct beliefs: the second character’s true belief about the actual situation and two false beliefs. The first-order FB question asks about the first character’s false belief about the situation (e.g., what does Sam think they are selling at the bake sale?). The second-order FB question asks about the second character’s false belief about the first character’s belief, and is broken down into two separate questions to avoid asking syntactically too complex questions (e.g., Maria is asked what Sam thinks they are selling at the bake sale, and then the child is asked what Maria will answer, thus effectively asking what Maria thinks that Sam thinks they are selling at the bake sale). See Hollebrandse et al. (2014, Appendix 1) for a sample item.

The task consisted of eight stories read to the child by the experimenter. Each story was accompanied by four pictures that were presented one by one on a computer screen. The task was divided in two blocks with a short break in between. The order of stories was counterbalanced across participants. Each story contained one second-order FB question and two first-order FB questions. The first first-order FB question was asked in the middle of the story, when the first false belief was introduced. At the end of the story, the second-order FB question was asked, followed by the first-order FB question. The first-order FB question was asked again at the end of the story in order to check whether children had difficulties with the length and complexity of the story.

One item was removed from further analysis since item analysis showed that the response on this item differed from the other seven items: on the second first-order FB question, mean accuracy on this item was only 0.48, while mean accuracy on other items varied between 0.79 and 0.92. Additionally, on this item, mean accuracy on the second-order FB question was higher (0.80) than on the easier first-order FB question (0.48). Inspection of this item revealed that its content differed from the other items in that an extra belief had inadvertently been introduced, which made the correct first-order FB answer less plausible. Two dependent measures were calculated: mean accuracy on the first first-order FB question (FB1) and mean accuracy on the second-order FB question (FB2).

#### Working Memory

Working memory is the ability to temporarily maintain and manipulate information (Baddeley and Hitch, 1974). It can be operationalized in different ways. Because of the known language and communication difficulties of children with ASD and children with ADHD, we wanted to reduce the verbal load of the working memory task by using a visual task, rather than a verbal task such as a listening span task or digit span task. Specifically, we operationalized working memory by the n-back task (Owen et al., 2005). The n-back task is a continuous performance task to measure working memory capacity. The task is commonly used in psychology and cognitive neuroscience (e.g., Williams et al., 2005; Cui et al., 2010; Chatham et al., 2011; de Vries and Geurts, 2014) and requires sustained maintenance and updating of information in working memory.

The n-back task in our study included three experimental conditions: 0-back (baseline), 1-back, and 2-back. In each condition, pictures were presented on a computer screen with a stimulus duration of 1000 milliseconds, followed by an interstimulus interval of 1500 milliseconds. In the 0-back condition, participants were instructed to press the yes-button when they saw a picture of a car, and to press the no-button when another picture appeared. In the 1-back condition, participants had to press the yes-button when the picture matched the picture immediately preceding it, and otherwise press the no-button. In the 2-back condition, participants had to press the yes-button when the picture matched the picture that appeared two pictures back, and otherwise press the no-button. Studies have shown that 2-back tasks seem suitable for children in our age range (e.g., Schleepen and Jonkman, 2010). The task was divided in different blocks, which were presented in random order. Each block started with 0-back, followed by 1-back and then 2-back. In this way, children got used to the task and were able to understand the more difficult 2-back condition. Participants started with a practice session of 15 trials per condition (0-, 1-, and 2-back), followed by the test session consisting of four blocks of 15 trials per condition (resulting in a total of 60 trials per condition). The total number correct on the 2-back condition was calculated as a measure of working memory.

#### Response Inhibition

The study also included a task to measure response inhibition. Response inhibition is the capacity to suppress an ongoing motor response that is no longer relevant. To capture response inhibition, we used a stop task, which is considered a relatively pure, reliable and valid measure of prepotent response inhibition (Tannock et al., 1989; Kindlon et al., 1995; de Vries and Geurts, 2014). Like the n-back task, the stop task is often used in psychology and cognitive neuroscience, and measures individual, clinical and developmental differences in the inhibition of responses. In this study we adopted the stop task from van den Wildenberg and Christoffels (2010). This is a non-verbal response inhibition task, which we preferred over a verbal task for the same reason as mentioned for the n-back task.

In this stop task, simple drawings of a tree and a door were presented on the computer screen. During go-trials, participants were asked to press the button corresponding with the picture on a two-button box. In 30% of the trials, a visual stop-signal was presented: a red square frame surrounding the picture border. When confronted with the stop-signal, participants had to inhibit the go-response by not pressing the button. The interval between the onset of the go-picture and the onset of the stop-signal (stop-signal delay) was set at 200 ms on the first stop-trial. An online tracking algorithm adjusted stop-signal delay as a function of individual stopping performance (Levitt, 1971). If the participant was able to stop, the stop-signal delay increased by 50 ms, thereby decreasing the chances of successful inhibition on the next stop-trial. After a failed-inhibition trial, the stop-signal delay decreased by 50 ms. This adaptive algorithm ensured successful inhibition on about 50% of the stop-trials, a procedure that yields reliable estimates of the Stop Signal Reaction Time (SSRT: Band et al., 2003). SSRT was calculated as a measure of response inhibition.

### Procedure

Children and their parents were recruited by brochures at schools and in outpatient clinics for child and adolescent psychiatry in Groningen. They took part in a larger study on language and communication in ASD and ADHD (Kuijper, 2016). The study was reviewed and approved by the research ethics committee CETO of the University of Groningen. Parents of all child participants gave written informed consent prior to participation in the study. Children and parents came to the lab together. Children were tested individually on a single day in a quiet testing room with two experimenters present. After every task children had a short break.

Two participants were excluded from further analysis: one (ASD) because he finished neither the comprehension task nor the production task, leaving 47 children in the ASD group, and the other (ADHD) because he scored below 0.75 on the control items in the comprehension task, leaving 36 children in the ADHD group. Furthermore, one child (ASD) conducted only half of the False Belief task and was removed from analyses involving this task. One child (ASD) did not finish the n-back task and was removed from analyses involving the n-back task. Another child (ADHD) did not complete the stop task and consequently was excluded from analyses involving this task. Finally, one child (ADHD) finished neither the n-back nor the stop task and was excluded from analyses including these tasks.

### Coding of Production Data

Children’s answers on the production task were voice-recorded. Only active transitive sentences containing a subject and an object that referred to one of the two animals in the picture were included in analyses (93.1% of all items). In the production task, more answers are acceptable than only object pronouns or reflexives. For pictures showing an other-oriented action, the use of a full noun phrase (e.g., “the elephant is hitting the crocodile”) to describe such actions is compatible with the binding principles. In fact, such a choice is pragmatically felicitous as well, as adults produce mainly full noun phases in this sentence context (see Spenader et al., 2009). Both the use of object pronouns (e.g., “the elephant is hitting him”) and the use of full noun phrases were therefore coded as correct responses in this condition. For pictures showing a self-oriented action, only the use of a reflexive (e.g., “the elephant is hitting himself”) was treated as accurate. All items were scored independently by two coders, who were blind to the participant’s diagnosis. The coders scored the grammatical form of the object (pronoun, reflexive, or full noun phrase). Inter-scorer agreement was high (Cohen’sκ = 0.95).

### Data Analysis

The data were analyzed using Generalized Linear Mixed Models (GLMM). A logit link was used to accommodate the repeatedly measured binary outcome variable (i.e., accuracy of pronoun interpretation, denoted below as Accuracy) (Jaeger, 2008; Heck et al., 2012). Compound symmetry was used as covariance matrix. First we tested for differences between groups in pronoun comprehension. Contrasts between diagnostic groups and controls (ASD vs. TD and ADHD vs. TD) were dummy-coded and included as fixed factors in the analysis. Whether the sentence matched the picture (coded as 0) or not (coded as 1) was additionally included as a fixed factor. This last factor was included because previous studies showed clear differences between match and mismatch conditions, likely caused by a yes-bias (see also Chien and Wexler, 1990; van Rij et al., 2010). In addition to these three main effects (denoted as ASD, ADHD, and Match) we included two two-way interactions (ASD^∗^Match, ADHD^∗^Match) in the model. A two-way interaction or main effect that had no effect on Accuracy (*p* > 0.05) was removed from the model.

Next, we examined possible cognitive mechanisms underlying object pronoun interpretation by including the relevant parameters derived from the False Belief task (FB1 and FB2), the n-back task (working memory, or WM), and the stop task (SSRT), respectively. All four were mean-centered around a value of zero and were included, in four separate analyses, as fixed factors in the aforementioned model. Interactions that had no effect on Accuracy (*p* > 0.05) were removed from the model. Finally, we tested whether found associations held up when all main and interaction effects with a significance value of *p* ≤ 0.05 were examined simultaneously in a multiple GLMM analysis.

## Results

### Pronoun and Reflexive Comprehension Task

In line with our expectations, neither the reflexive match condition nor the reflexive mismatch condition yielded a substantial number of errors (see Table 2). Therefore we did not statistically test for differences in reflexive interpretation between the groups. Below, our focus is on the two object pronoun conditions. Despite the rather small differences in performance in the object pronoun conditions, there was enough variance to build a meaningful GLMM.

**TABLE 2:** Mean proportions correct responses and standard deviations per group and per condition in the comprehension task.

	**Object pronoun match**		**Object pronoun mismatch**		**Reflexive match**		**Reflexive mismatch**	
	**Mean**	***SD***	**Mean**	***SD***	**Mean**	***SD***	**Mean**	***SD***
TD	0.98	0.07	0.87	0.29	0.99	0.04	1	0.00
ASD	0.98	0.07	0.84	0.28	0.99	0.05	0.99	0.05
ADHD	0.94	0.12	0.75	0.34	0.97	0.08	0.99	0.04

#### Clinical Groups

As expected (Chien and Wexler, 1990; van Rij et al., 2010), a significant effect of Match was found (see Table 3), indicating that more errors were made in the object pronoun mismatch condition than in the object pronoun match condition. Interactions of ASD or ADHD with Match did not contribute significantly to participants’ scores on the comprehension task (all *p*-values > 0.05), showing that this effect held for all groups. In addition, the main effects of ASD and ADHD did not significantly contribute to Accuracy.

**TABLE 3:** Estimated effects for Clinical group and Match on accuracy in object pronoun interpretation.

**Predictor**	**Estimate**	***SE***	***p***
Match	−1.94***	0.28	<0.001
ASD vs. TD	−0.26	0.50	0.60
ADHD vs. TD	−0.79	0.50	0.11

*p < 0.05; **p < 0.01; ***p < 0.001.

With no differences among the groups, we conclude that errors in object pronoun interpretation are not explained by the presence of ASD or ADHD. In subsequent analyses, main and interaction effects related to diagnostic group were removed, leaving a model that included two main effects (Mechanism and Match) and one interaction effect (Mechanism^∗^Match).

Because the TD group differs from the ASD and ADHD group in mean IQ-score and the TD group differs from the ADHD group in mean PPVT-score (see Table 1), we checked post-hoc if group differences in pronoun interpretation between ASD, ADHD and TD emerge, by (i) selecting part of our TD group (*n* = 27) to match the IQs of both other groups, and by (ii) selecting part of our TD group (*n* = 34) to match the PPVT of the ADHD group. No group differences in pronoun interpretation emerge when we use the subgroups matched on IQ or verbal ability in the two *post hoc* analyses (see Table 4).

**TABLE 4:** Estimated effects for Clinical group and Match on accuracy in object pronoun interpretation for the IQ-matched subgroup and for the PPVT-matched subgroup.

	**IQ-matched subgroup (*n* = 110)**			**PPVT-matched subgroup (*n* = 117)**		
**Predictor**	**Estimate**	***SE***	***p***	**Estimate**	***SE***	***p***
Match	–1.97***	0.30	<0.001	1.94***	0.28	<0.001
ASD vs. TD	–0.13	0.51	0.81	–0.14	0.50	0.79
ADHD vs. TD	–0.67	0.51	0.19	–0.67	0.50	0.18

*p < 0.05; **p < 0.01; ***p < 0.001.

#### Mechanisms

No interaction effect of Match with any of the cognitive mechanisms was found (all *p*-values > 0.05). Therefore, in the final model only the main effects of each of the cognitive mechanisms and Match were included, first separately, and next in the multiple GLMM. We found a main effect of FB2 (see Table 5). Lower scores on second-order False Belief questions were associated with lower Accuracy scores in both the Object pronoun match and the Object pronoun mismatch condition. We also found a significant main effect of SSRT. Higher SSRT scores (indicating lower inhibition) were associated with more errors in the object pronoun conditions. No significant effects of FB1 or working memory were found. In all four analyses, the main effect of Match remained significant: more errors were made in the object pronoun mismatch condition than in the object pronoun match condition.

**TABLE 5:** Estimated effects of Mechanism and Match on accuracy in object pronoun interpretation.

	**FB1**			**FB2**			**SSRT**			**WM**		
**Predictor**	**Estimate**	***SE***	***p***	**Estimate**	***SE***	***p***	**Estimate**	***SE***	***p***	**Estimate**	***SE***	***p***
Match	−2.00***	0.30	<0.001	−2.02***	0.30	<0.001	−2.05***	0.30	<0.001	−1.99***	0.29	<.001
Mechanism	1.71	1.00	0.085	1.23*	0.51	0.017	−0.006**	0.002	0.008	0.031	0.02	0.13

*p < 0.05; **p < 0.01; ***p < 0.001.

FB2, SSRT and Match were included in a multiple GLMM (Table 6). All aforementioned associations remained significant. Thus, when adjusted for the effect of SSRT, lower scores on FB2 questions were still associated with lower Accuracy scores in the object pronoun conditions. Vice versa, when adjusted for the effect of FB2, higher SSRT scores were still associated with lower Accuracy scores in the object pronoun conditions. Furthermore, a main effect for Match remained: adjusted for the effects of FB2 and SSRT, children still performed worse in the object pronoun mismatch condition than in the object pronoun match condition.

**TABLE 6:** Estimated multiple mechanisms model of accuracy in object pronoun interpretation.

	**Estimate**	***SE***	***p***
Intercept	3.72***	0.31	<0.001
Match	−2.07***	0.31	<0.001
FB2	1.13*	0.50	0.025
SSRT	−0.005*	0.002	0.018

Only main effects and interactions *p* < 0.05 in univariate analyses were included in multiple analyses. *p < 0.05; **p < 0.01; ***p < 0.001.

In a *post hoc* analysis we added age to our model. In our study we focused on children in the age range of 6–12 years, during which the Pronoun Interpretation Problem gradually disappears. With age being associated with FB2 and SSRT, age was added to our model to study the extent to which age would subsume the effects of FB2 and SSRT.

Table 7 shows that the effects of FB2 and SSRT were attenuated when age was included, confirming that children’s pronoun interpretation errors decrease with age and indicating that age is more strongly linked to object pronoun interpretation than theory of mind and inhibition. The main effect of Match remained significant: children made more pronoun interpretation errors in the mismatch condition than in the match condition.

**TABLE 7:** Estimated multiple mechanisms model of accuracy in object pronoun interpretation, including Age.

	**Estimate**	***SE***	***p***
Intercept	1.41	0.93	0.13
Match	−2.10***	0.31	<0.001
FB2	0.82	0.53	0.12
SSRT	−0.004	0.002	0.07
Age	0.02**	0.008	0.008

Only main effects and interactions *p* < 0.05 in univariate analyses were included in multiple analyses. *p < 0.05; **p < 0.01; ***p < 0.001.

### Pronoun and Reflexive Production Task

In production, consistent with our expectations, children hardly made any mistakes (see Table 8). With all three groups performing at ceiling, we did not test for group differences in production accuracy.

**TABLE 8:** Mean proportions correct responses in the production task.

	**Other-oriented action**		**Self-oriented action**	
	**Mean**	***SD***	**Mean**	***SD***
TD	1.0	0.00	1.0	0.06
ASD	0.99	0.11	0.99	0.08
ADHD	1.0	0.00	0.99	0.12

Recall that, for the other-oriented action, both the use of an object pronoun and the use of a full noun phrase were scored as correct responses. Only in 5% of the cases an object pronoun was used. In the remaining 95% of the cases a full noun phrase was used. This corresponds with the pattern of production displayed by Dutch adults, who also mainly used full noun phrases to describe an other-oriented action in a similar experiment (Spenader et al., 2009). Importantly, children hardly ever incorrectly use an object pronoun [4 out of 769 scorable sentences, produced by three children (two ADHD and one ASD)] or a reflexive [4 out of 794 scorable sentences, produced by only one child (ASD)].

We tested, *post hoc*, if children with ASD or ADHD differed from TD children in their use of full noun phrases and object pronouns. A GLMM was performed on all items in the other-oriented condition, with full noun phrase (yes or no) as binary dependent variable and two dummy-coded contrasts between diagnostic groups and controls (ASD vs. TD and ADHD vs. TD) as fixed factors. No significant differences between the groups were found (all *p*-values > 0.05): children with ASD used a full noun phrase in 96% of the cases, children with ADHD in 95% of the cases and TD children in 94% of the cases. This indicates that children with ASD and children with ADHD use the same linguistic forms as TD children to express other-oriented and self-oriented actions.

## Discussion

The aim of this study was to clarify how children acquire object pronoun interpretation and production by investigating the possible cognitive mechanisms underlying the Pronoun Interpretation Problem, as different theoretical accounts see a role for different cognitive mechanisms. We found that both second-order False Belief performance and Stop Signal Reaction Time were associated with performance on the object pronoun interpretation task. These results suggest that theory of mind and inhibition are necessary for object pronoun interpretation. This finding is compatible with the perspective taking account of the Pronoun Interpretation Problem by Hendriks and Spenader (2006). According to Hendriks and Spenader’s account, object pronouns are potentially ambiguous and listeners must consider the perspective of the speaker to block the incorrect coreferential interpretation for the object pronoun. The results of this study suggest that the Pronoun Interpretation Problem arises if children fail to consider the perspective of the speaker because of insufficient theory of mind abilities, or fail to suppress the incorrect interpretation of the pronoun because of poor inhibition skills.

We did not find a relation between working memory and performance on object pronoun interpretation and thus found no support for Reinhart’s (2006, 2011) claim that sufficient working memory is necessary for the costly operation of reference-set computation that is needed for object pronoun interpretation. The absence of a relation with working memory corroborates the results of Perovic and Wexler (2018). They found that children with Williams Syndrome, who are generally reported not to have memory deficits, nevertheless showed difficulties with pronoun interpretation in simple transitive sentences in English. However, these children did not receive a working memory task to confirm that they did not have memory deficits. Contrasting with these findings, in children with Developmental Language Disorders (DLD) Montgomery and Evans (2009) found a relation between working memory, as measured by a listening span task, and performance on the interpretation of complex sentences, including embedded pronominal sentences such as “Bugs Bunny says Daffy Duck is hugging him.” However, performance on different sentence types was combined in this study and also included performance on embedded reflexive sentences and passive sentences. Additionally, the embedded pronominal sentences in this study were more complex than the simple transitive pronominal sentences in the current study (see also Ladányi et al., 2017, who found a relation in children with DLD between performance on the n-back task and the interpretation of pronouns and reflexives in embedded sentences in Hungarian). Because of these differences with the current study, it is possible that the relation with working memory reported in previous studies with children with DLD is due to other features of the linguistic materials than the presence of object pronouns, for example the syntactic complexity of the test sentences used. This explanation is supported by the close link found between working memory capacities and complex syntax in children’s comprehension of language, as measured with different working memory tasks and different syntactic constructions (Delage and Frauenfelder, 2019).

Regarding children’s production of pronouns and reflexives, we did not find support for Chien and Wexler’s (1990) pragmatic explanation of the Pronoun Interpretation Problem, as the children hardly made any binding errors in their production of object pronouns or reflexives. That is, they rarely produced a reflexive when a pronoun or full noun phrase was the correct form to use (which would constitute a violation of Principle A), and they rarely produced a pronoun when a reflexive was the correct form to use (which would constitute a violation of Principle B, in generative syntactic terms). Thus, the children observed the constraints of the grammar in their production of these forms. This finding is in line with previous experiments with typically developing children, showing that children produce object pronouns in an adult-like way from a young age (De Villiers et al., 2006; Matthews et al., 2009; Spenader et al., 2009).

Because of their known difficulties with theory of mind, working memory, and inhibition, we had expected children with ASD and children with ADHD to have more problems with object pronoun interpretation than TD children. However, we did not find any differences in object pronoun interpretation between children with ASD, children with ADHD, and TD children: all three groups made errors in object pronoun interpretation. As expected, we also found that all three groups performed at ceiling on the reflexive conditions and on the production task. That is, the TD children as well as the children with ASD or ADHD in our study only had problems with the interpretation of object pronouns (particularly emerging in the mismatch condition), and did not have difficulty with the interpretation of reflexives (either in the match condition or in the mismatch condition) or with the production of pronouns and reflexives.

That children with ASD and TD children show a similar Pronoun Interpretation Problem corroborates the findings by Perovic et al. (2013). Perovic et al. (2013) consider the Pronoun Interpretation Problem to be pragmatic in nature (cf. Chien and Wexler, 1990). At first glance, this leaves unexplained why they did not find differences between children with ASD and TD children in object pronoun interpretation. After all, if the Pronoun Interpretation Problem is a pragmatic problem, why would children with ASD, who are known for their pragmatic deficits, not make more errors in object pronoun interpretation than TD children?

Perovic et al. (2013) argue that there may be different kinds of pragmatics: a kind of pragmatics related to social rules and a kind of pragmatics more directly related to language (cf. Schaeffer, 2003). This latter so-called “linguistic pragmatics” may not be affected in ASD, according to Perovic et al. (2013). We propose an alternative explanation of these findings. Rather than positing two types of pragmatics, one of which is unaffected in ASD, we propose that perspective taking need not be a pragmatic process but can also be part of the grammar. According to Hendriks and Spenader’s (2006) account of the Pronoun Interpretation Problem, the interpretation of an object pronoun requires listeners to take into account the perspective of a hypothetical speaker in order to determine the interpretation of the pronoun (see also Hendriks, 2014). That is, listeners must apply the relevant constraints of the grammar to determine the optimal meaning of the pronoun, and must additionally place themselves in the perspective of a hypothetical speaker and apply the same constraints to determine the optimal form for this optimal meaning. In a final step, the listener must check whether the input form in comprehension and the output form in production match, or in other words: whether a speaker would have used a pronoun to express the selected interpretation. If so, the selected interpretation is considered to be correct, but if not, the selected interpretation must be suppressed and another interpretation must be checked. This process of “grammaticalized perspective taking,” which requires listeners to take the perspective of a hypothetical speaker and express a particular meaning as if they were the speaker, may be different from taking the perspective of an actual speaker, who may or may not be sitting in front of the listener. The latter form of perspective taking is much more challenging for listeners, since it differs per speaker and per situation. In contrast, grammaticalized perspective taking may be less demanding, as it does not vary per situation and therefore could be gradually automatized (as is shown in computational cognitive simulations to be psychologically plausible, see van Rij et al., 2010; Vogelzang et al., 2021). Such an automatized process can be understood as being part of the grammar of a mature native speaker.

This view of object pronoun interpretation as a process of grammaticalized perspective taking is supported by the finding of similar difficulties with pronoun interpretation in non-advanced second-language learners as in children acquiring their native language. The finding of a Pronoun Interpretation Problem in second-language learners has been put forward as evidence in favor of Reinhart’s costly operation of reference-set computation and against an explanation in terms of lack of linguistic knowledge (Slabakova et al., 2017), but is also consistent with the proposed computationally complex process of perspective taking. These results may thus provide support for the claim that this grammaticalized perspective taking is unaffected in children with ASD and ADHD. This corroborates previous findings of similar linguistic performance in ASD children, ADHD children, and TD children (Kim and Kaiser, 2000; Geurts et al., 2004a; Geurts and Embrechts, 2008; Helland et al., 2012). In contrast, taking the perspective of an actual speaker may be involved in pragmatic skills such as turn-taking and conversational rapport, both of which are found to be impaired in ASD and ADHD (e.g., Geurts et al., 2004a; Green et al., 2014).

Most of the ASD children in our study could be classified as “language normal” (based on their PPVT scores and the vocabulary subtest of the WISC-III, cf. Kjelgaard and Tager-Flusberg, 2001). Perovic et al. (2013) found that the linguistic performance of ASD children with language impairment differed from the linguistic performance of ASD children without language impairment. However, they only found differences in the interpretation of reflexives, while both groups of ASD children performed similarly on the interpretation of object pronouns. A crucial difference between the study of Perovic et al. (2013) and the present study is the type of task that is used. Perovic et al. (2013) used a Picture Selection Task, which tests for preference of interpretation, whereas our study used a Picture Verification Task, which tests for acceptability of interpretation. On the basis of the study of Perovic et al. (2013) it can be concluded that ASD children with language impairment have a preference for a non-coreferential interpretation for object pronouns and reflexives. However, it is not clear whether these children would incorrectly accept a coreferential interpretation for pronouns, which is what the Pronoun Interpretation Problem entails. To further unravel differences between ASD children with and without language impairment, such children could be tested on their interpretation of object pronouns and reflexives using a Picture Verification Task or some other task testing acceptability rather than preference for one of the two relevant interpretations.

A finding of our study that may at first sight be surprising is the fact that we found an effect of second-order False Belief understanding, but no effect of first-order False Belief understanding. The absence of an association between first-order False Belief understanding and object pronoun interpretation is probably due to ceiling performance in first-order False Belief understanding (see Table 1). First-order False Belief understanding is generally mastered around age 4, so at least 2 years before object pronouns are understood correctly (De Villiers et al., 2006). Because we expected a ceiling effect for first-order False Belief understanding in our 6- to 12-year-old children, we included a task that also measured second-order False Belief understanding. Since accurate second-order False Belief understanding is dependent on accurate first-order False Belief understanding, a slower development of first-order theory of mind is expected to result in a slower development of second-order theory of mind as well, thus allowing us to investigate the relation between pronoun interpretation and theory of mind by looking at second-order False Belief understanding. Second-order False Belief understanding was found to relate to object pronoun interpretation, which indicates that perspective taking is important in interpreting object pronouns.

The False Belief task used in this study is a highly verbal task, which also depends on general language skills. Therefore, it could be argued that the observed relation between False Belief understanding and performance on pronoun comprehension merely reflects children’s general language abilities. However, if true, we would expect this to also be reflected in children’s performance on reflexive comprehension. The reflexive condition can be considered a control condition, assessing children’s general language comprehension abilities and, more specifically, their syntactic abilities. Since the children in our study did not have any problems in the reflexive condition, their general language comprehension abilities appear to be intact. Previous studies found significant relations between various aspects of language and False Belief understanding (for an overview, see Milligan et al., 2007). Our study adds to this the observation of a relation between object pronoun interpretation and False Belief understanding. Yet it would be worthwhile to examine the relation between object pronoun interpretation and theory of mind using other theory of mind tasks, for example low-verbal theory of mind tasks or the (more natural) strange stories task (Happé, 1994).

Although we did not find an association between working memory and performance in object pronoun interpretation, it should be kept in mind that working memory is a broad concept and many different tasks for its measurement have been developed. In our study, an n-back task with non-verbal stimuli (pictures) was used. It is possible that working memory tasks with verbal stimuli are associated with object pronoun interpretation. However, meta-analyses show that both working memory tasks with verbal stimuli and with non-verbal stimuli relate to general language comprehension (Daneman and Merikle, 1996) and that both give rise to similar activation patterns in neuroimaging studies (Owen et al., 2005). Additionally, in a related study with the same children (Kuijper et al., 2015), a relation was found between performance on the n-back task with non-verbal stimuli and performance on another linguistic task than the one reported on here. This other linguistic task tested speakers’ referential choice between using a pronominal subject and using a full noun phrase subject in production, which is dependent on how well the speaker can keep track of the different referents mentioned in the preceding linguistic discourse. This indicates that the n-back task used in this study relates to at least some aspect of linguistic performance that requires working memory. Since no association was found between the n-back task and performance on object pronoun interpretation in the present study, this strongly suggests that object pronoun interpretation and working memory are unrelated.

In contrast to working memory, inhibition was found to be associated with object pronoun interpretation in our study. In our study, we used a stop task to measure prepotent response inhibition. Yet, it may be worthwhile to also investigate the relation between pronoun interpretation and other types of inhibition, in particular interference control (i.e., cognitive inhibition). A final consideration with regard to the cognitive processes that were studied here pertains to the role of age. In a *post hoc* analysis we added age to our final model, leading to attenuation of the effects of inhibition and theory of mind. Age, as the umbrella variable, was more strongly linked to object pronoun interpretation than the specific effects of theory of mind and inhibition. The effect of age shows that, in addition to theory of mind and inhibition, other cognitive factors are likely involved in pronoun interpretation which also develop with age and which we have not included in this study. These cognitive factors (i.e., the included as well as non-included ones) are all subsumed by the overarching factor of age. Although the theoretical literature on object pronoun interpretation is not explicit about this, possibly cognitive flexibility (to switch from the incorrect interpretation to the alternative correct interpretation, cf. Kissine, 2012) or focused attention (to process speech in real-time, see, e.g., Wolfgramm et al., 2016) play a role too.

In summary, the current study provides insight into the Pronoun Interpretation Problem and the cognitive mechanisms underlying this comprehension delay in children’s language development. We found that both theory of mind and inhibition skills were associated with performance on object pronoun interpretation. This provides support for Hendriks and Spenader’s (2006) perspective taking account of object pronoun interpretation, which holds that listeners must take into account the perspective of a hypothetical speaker and thus block the incorrect interpretation for the pronoun. Furthermore, our study showed that the performance of children with ASD or ADHD was comparable to that of TD children: the three groups demonstrated similar difficulties in their interpretation of object pronouns and neither of the groups showed difficulties in the production of object pronouns and reflexives. This suggests that children with ASD and children with ADHD do not have more problems than TD children in taking into account the grammatical perspective of a hypothetical speaker, despite their possible difficulties in perspective taking with actual conversational partners.

## Data Availability Statement

The raw data supporting the conclusions of this article will be made available by the authors, without undue reservation.

## Ethics Statement

This study, involving human participants, was reviewed and approved by the Commissie Ethische Toetsing Onderzoek (CETO) of the University of Groningen. Written informed consent to participate in this study was provided by the participants’ legal guardian/next of kin.

## Author Contributions

SK, CH, and PH designed the study. SK carried out the experiment. SK, CH, and PH analyzed the results and wrote the manuscript. All the authors read and approved the final version of the manuscript.

## Conflict of Interest

The authors declare that the research was conducted in the absence of any commercial or financial relationships that could be construed as a potential conflict of interest.
